# Synergistic Effects of nZVI and KH_2_PO_4_ on Phenolic Accumulation, Antioxidant Capacity and Fruit Quality of Marselan Grape via Multi-Omics

**DOI:** 10.3390/plants15111595

**Published:** 2026-05-22

**Authors:** Guangling Shi, Baozhen Zeng, Yu Li, Huimin Gou, Shixiong Lu, Xiaoying Wu, Guoping Liang, Baihong Chen, Juan Mao

**Affiliations:** College of Horticulture, Gansu Agricultural University, Lanzhou 730070, China; 19158253696@163.com (G.S.); 1073325010148@st.gsau.edu.cn (B.Z.); liyu082417@163.com (Y.L.); 1648885861@163.com (H.G.); 14401@gsau.edu.cn (S.L.); 13625016240@163.com (X.W.); lianggp@gsau.edu.cn (G.L.);

**Keywords:** *Vitis vinifera* cv. Marselan, nZVI, KH_2_PO_4_, fruit quality, transcriptomics, metabolomics, flavonoid biosynthesis

## Abstract

Precision nano-fertilization offers transformative potential for sustainable improvement of grape quality, yet the underlying molecular mechanisms remain poorly understood. Here, we investigated the effects of foliar-applied nano zero-valent iron (nZVI) and potassium dihydrogen phosphate (KH_2_PO_4_), in combination, on berry quality and secondary metabolic reprogramming in *Vitis vinifera* cv. Marselan. The combined nZVI/KH_2_PO_4_ treatment improved photosynthetic capacity, Fe/P co-accumulation, and berry quality traits including soluble solid content, sugar–acid ratio, and phenolic and aroma metabolite profiles. Crucially, integrated transcriptomic and metabolomic profiling identified 631 differentially expressed genes and 838 differentially accumulated metabolites, converging on flavonoid biosynthesis and glutathione metabolism as the dominant regulatory axes. Correlation network analysis pinpointed five hub regulatory genes—*VvHCT*, *VvFLS1*, *VvLAR1/2*, *VvUGT88F5*, and *VvODC*—as central orchestrators of nanomaterial-driven metabolic reprogramming: *VvHCT* and *VvFLS1* coordinately redirected carbon flux toward hydroxycinnamic acid conjugates and flavonol accumulation, while *VvLAR1/2* governed proanthocyanidin polymerization, and *VvUGT88F5* modulated glycosylation-dependent metabolite stabilization. Notably, *VvODC* linked polyamine metabolism to glutathione-mediated stress buffering, revealing a previously uncharacterized crosstalk between nano-iron signaling and antioxidant reprogramming. These findings establish a mechanistic framework in which nZVI and KH_2_PO_4_ synergistically remodel the secondary metabolome through discrete yet interconnected transcriptional nodes, providing molecular targets for nano-enabled precision viticulture and broader applications of engineered nanomaterials in high-value crop improvement.

## 1. Introduction

Grapes (*Vitis vinifera* L.) rank among the most economically significant fruit crops globally, serving as indispensable raw materials for the wine, food, and nutraceutical industries [1]. Beyond their longstanding role in winemaking, grape berries have gained increasing recognition as a rich reservoir of bioactive compounds—including flavonoids, anthocyanins, condensed tannins, and phenolic acids—that confer potent antioxidant activity and offer well-documented benefits to human health [2]. These metabolites not only govern the sensory and organoleptic characteristics of grapes and derived wines but also represent high-value functional ingredients of considerable industrial importance [3]. Consequently, strategies aimed at enhancing the coordinated accumulation of these compounds are central to improving both intrinsic fruit quality and the commercial competitiveness of grape-based products.

Among premium red wine cultivars, Marselan—a hybrid of ‘Cabernet Sauvignon’ and ‘Grenache Noir’—has attracted growing interest owing to its broad adaptability, including tolerance to drought and fungal diseases, combined with outstanding enological potential [4,5]. This cultivar is now extensively cultivated across major wine-producing regions, including China, France, and Spain. The Hexi Corridor in northwestern China, in particular, offers favorable climatic and edaphic conditions for premium viticulture and has emerged as one of the most promising new wine-producing zones in Asia [6,7]. Nevertheless, achieving consistent and reproducible improvements in fruit quality—particularly with respect to phenolic composition, sugar–acid balance, and aroma profile—remains a central challenge for producers seeking to strengthen the industrial competitiveness and market value of Marselan wines.

Foliar fertilization represents an effective and environmentally compatible agronomic strategy for rapidly modulating plant nutritional status and metabolic activity. Prior research has demonstrated that foliar nitrogen supplementation from veraison to harvest can significantly promote the accumulation of flavonols, fatty acids, and volatile aroma compounds in grape berries [8,9]. As demand intensifies for functionally enriched and high-quality agricultural produce, the development of precision foliar fertilization strategies has become a research priority in contemporary viticulture [10]. In this context, nZVI has emerged as a particularly promising candidate for agricultural application, owing to its distinctive physicochemical properties, including high surface reactivity, superior bioavailability, and nanoscale iron release kinetics [10,11]. Enhanced iron availability facilitates chlorophyll biosynthesis and photosynthetic efficiency—two foundational processes governing carbon assimilation and downstream metabolite production [12,13,14,15]. Furthermore, iron-based nanomaterials have been reported to improve fruit quality attributes and postharvest performance by optimizing mineral nutrient status and suppressing plant-pathogenic microorganisms [16,17,18]. KH_2_PO_4_, a widely adopted phosphorus–potassium fertilizer, plays pivotal roles in cellular energy metabolism, phloem sugar transport, and the biosynthesis of secondary metabolites [19,20,21,22]. Foliar delivery of KH_2_PO_4_ has been shown to elevate soluble sugar content, optimize the sugar–acid ratio, and promote the accumulation of phenolic compounds including anthocyanins [23,24]. Recent studies have further advanced our understanding in this area: a 2025 study on ‘Marselan’ grapevines in the Wuwei region of the Hexi Corridor demonstrated that foliar application of nZVI and KH_2_PO_4_ enhanced leaf photosynthetic parameters and mineral nutrient content [25], in parallel, a 2024 study identified *VviMYB24* as a key positive regulator of flavonol biosynthesis in grapevine under moderate drought stress [26].

Despite these advances, three critical knowledge gaps remain unaddressed: Gap 1: Uncharacterized nZVI-KH_2_PO_4_ synergy. Whether combined application produces synergistic effects on the coordinated accumulation of flavonoids, anthocyanins, and aroma metabolites remains unknown. Gap 2: Undefined source-to-sink metabolic linkages. The causal pathways through which nano-fertilization-enhanced photosynthesis and carbon allocation drive targeted flux into phenolic and aroma biosynthesis pathways in developing berries remain poorly resolved. Gap 3: Absence of integrated multi-omics characterization. The transcriptional regulatory nodes governing secondary metabolic reprogramming under nano-fertilization, and their functional associations with target metabolite accumulation, have not been systematically identified. To address these gaps, we evaluated the individual and combined effects of foliar-applied nZVI and KH_2_PO_4_ on berry quality in *V. vinifera* cv. Marselan, integrating physiological, metabolomic, and transcriptomic approaches to elucidate the mechanisms underlying nano-fertilization-driven enhancement of grape functional quality.

## 2. Results

### 2.1. Effects of nZVI and KH_2_PO_4_ on Leaf Source Function

#### 2.1.1. Leaf Morphology and Anatomical Structure

**Figure 1:**
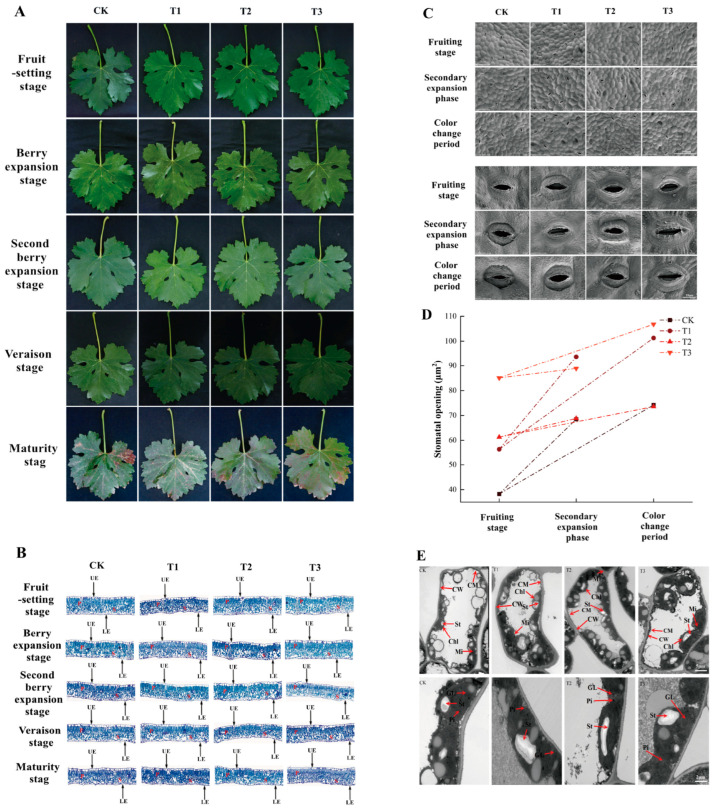
Foliar Application of Nanoscale Zero-Valent Iron, Potassium Dihydrogen Phosphate, and Their Combination Alters Leaf Phenotype, Anatomical Structure, Stomatal Traits, and Mesophyll Cell Ultrastructure of Marselan Grape (*Vitis vinifera* L. cv. Marselan) During Key Growth Stages. (**A**) Phenotypic characteristics of grape leaves at five critical growth stages (fruit-setting stage, berry expansion stage, second berry expansion stage, veraison stage, and maturity stage) under different treatments. (**B**) Light micrographs of cross-sectional anatomical structure of grape leaves at each corresponding growth stage under different treatments. (**C**) Light micrographs of leaf epidermal cell morphology (upper panel) and stomatal morphology (lower panel) at the fruit-setting stage, second berry expansion stage, and veraison stage under different treatments. (**D**) Dynamic changes in stomatal aperture (unit: μm^2^) of grape leaves at the three above-mentioned growth stages under different treatments. (**E**) Transmission electron microscopy (TEM) images showing the ultrastructure of grape leaf mesophyll cells under different treatments. Abbreviations for leaf anatomical structures: UE, upper epidermis, LE, lower epidermis. Abbreviations for subcellular structures: CW, cell wall, CM, cell membrane, Chl, chloroplast, Mt, mitochondrion, St, starch grain, GL, osmiophilic granule, SL, stroma lamella, Pi, plastoglobulus (lipid droplets within chloroplasts). The error bar represents the standard deviation (SD), the same below.

The combined application of nZVI and KH_2_PO_4_ markedly improved leaf structural integrity, with effects most pronounced at the fruit-set stage (Figure 1A,B; Table 1). Leaves under the combined treatment (T3) displayed a conspicuously darker green phenotype relative to controls, indicative of enhanced chlorophyll accumulation and augmented photosynthetic capacity. Quantitative anatomical measurements revealed that palisade tissue thickness and total leaf thickness increased by 9.16% and 8.41%, respectively, compared with the control, consistent with improved light interception efficiency and carbon assimilation potential. These structural adaptations are in agreement with previous reports demonstrating that iron and phosphorus co-supplementation promotes mesophyll cell expansion and tissue compactness in horticultural crops.

Microscopic examination further demonstrated that the upper and lower epidermis became more compact, while palisade cells were more elongated and orderly arranged under T3, collectively reflecting structural optimization favorable to photosynthetic performance. Although inter-treatment differences in anatomical parameters were less pronounced during intermediate developmental stages, overt senescence symptoms—including chlorosis and tissue loosening—were evident in control and single-treatment leaves during veraison and ripening. By contrast, the combined treatment sustained leaf structural integrity and substantially delayed senescence-associated deterioration. Notably, stomatal aperture increased by 40% at veraison under T3, facilitating CO_2_ diffusion and gas exchange efficiency. Transmission electron microscopy further revealed that chloroplast ultrastructure was better preserved under the combined treatment, with well-organized thylakoid membrane stacking and conspicuously increased starch granule deposition. Taken together, these structural improvements provide a robust physiological basis for enhanced assimilate production, which is a prerequisite for the downstream biosynthesis of functional metabolites with significant industrial relevance.

#### 2.1.2. Photosynthetic Characteristics During the Growing Season

**Figure 2:**
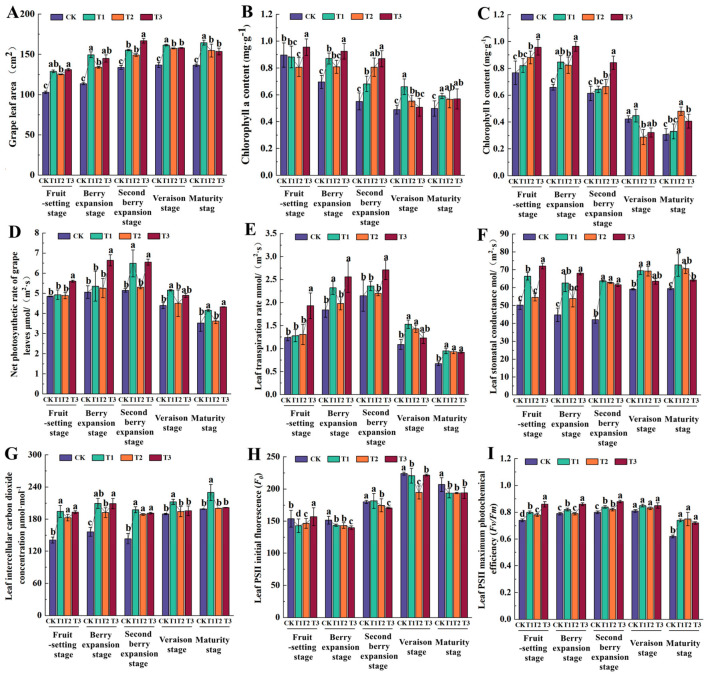
Effects of foliar application of nZVI, KH_2_PO_4_, and their combination on leaf growth, photosynthetic pigment content, gas exchange parameters, and chlorophyll fluorescence characteristics of *Vitis vinifera* cv. Marselan across five standardized growth stages: fruit-setting (Stage I), berry expansion (Stage II), second berry expansion (Stage III), veraison (Stage IV), and maturity (Stage V). (**A**) Leaf area (cm^2^). (**B**) Chlorophyll a content (mg·g^−1^ FW). (**C**) Chlorophyll b content (mg·g^−1^ FW). (**D**) Net photosynthetic rate, *Pn* (μmol·m^−2^·s^−1^). (**E**) Transpiration rate, *Tr* (mmol·m^−2^·s^−1^). (**F**) Stomatal conductance, *Gs* (mol·m^−2^·s^−1^). (**G**) Intercellular CO_2_ concentration, *Ci* (μmol·mol^−1^). (**H**) Minimum fluorescence of PSII, *F*_0_. (**I**) Maximum photochemical efficiency of PSII, *Fv*/*Fm*. Different lowercase letters above bars indicate significant differences among treatments at the same growth stage (*p* < 0.05, Duncan’s multiple range test). Different lowercase letters above the bars indicate significant differences among different treatments at the same growth stage (*p* < 0.05, Duncan’s multiple range test). The same below.

Consistent with the observed anatomical improvements, the combined treatment (T3) significantly enhanced photosynthetic performance across five standardized growth stages: fruit-setting (Stage I), berry expansion (Stage II), second berry expansion (Stage III), veraison (Stage IV), and maturity (Stage V) (Figure 2A–I). Leaf area and total chlorophyll (a + b) content were consistently superior to those of the control at all five stages, with peak increases of 22.9% and 23.5%, respectively, recorded at veraison (Stage IV). Gas exchange measurements further corroborated these trends: net photosynthetic rate (*Pn*), stomatal conductance (*Gs*), and transpiration rate (*Tr*) all peaked at veraison (Stage IV) and were significantly elevated under T3, with respective increases of 18.6%, 32.3%, and 19.8% relative to the control. Concurrently, intercellular CO_2_ concentration (*Ci*) exhibited an inverse pattern at Stage IV, indicating more efficient CO_2_ utilization and carboxylation activity. Importantly, Pn remained 39.9% higher than that of the control at maturity (Stage V), demonstrating the capacity of the combined treatment to sustain photosynthetic activity well into fruit maturation—a critical period for the synthesis and accumulation of berry quality compounds. Chlorophyll fluorescence analysis revealed a consistently higher *Fv*/*Fm* ratio and lower minimum fluorescence (*F*_0_) under T3 across all five stages, indicating improved photochemical efficiency and reduced susceptibility to photoinhibition. These findings are consistent with previous studies reporting that nano-enabled iron supplementation enhances the structural integrity of photosystem II reaction centers and promotes electron transport chain efficiency. Collectively, the combined application of nZVI and KH_2_PO_4_ reinforces photosynthetic source capacity and carbon assimilation throughout the entire fruit development period, thereby establishing a robust physiological foundation for the downstream accumulation of sugars and bioactive metabolites that collectively determine fruit quality and industrial value.

#### 2.1.3. Changes in Carbon Metabolism and Correlation with Photosynthetic Traits

The enhanced photosynthetic performance under T3 was accompanied by significant stimulation of carbon metabolic flux and nutrient accumulation (Figure S1A–K). Soluble protein, total soluble sugars, and starch contents followed a characteristic developmental trajectory, peaking at veraison, but were consistently and significantly elevated under the combined treatment relative to controls. At ripening, these parameters were increased by 13.6%, 28.6%, and 35.2%, respectively, confirming sustained carbon allocation and assimilate retention. Individual sugar profiling revealed marked increases in fructose, glucose, and sucrose, reflecting enhanced phloem loading, transport efficiency, and sink-directed carbon partitioning. Furthermore, the activities of key sucrose metabolism enzymes—including neutral invertase (NI), sucrose phosphate synthase (SPS), acid invertase (AI), and sucrose synthase (SS)—were significantly elevated under T3, indicating accelerated sucrose synthesis, interconversion, and turnover. These enzymatic responses are consistent with a model wherein nZVI-mediated improvement in iron bioavailability stimulates metabolic enzyme activity through cofactor-dependent mechanisms. Collectively, these results demonstrate that the combined treatment effectively amplifies source capacity and promotes assimilate accumulation, supplying essential carbon skeletons and energy equivalents for the biosynthesis of phenolics, flavonoids, and other functional metabolites. This enhanced carbon flux is of particular importance for improving the nutritional profile and industrial quality of grape berries destined for premium wine production. To explore links between leaf photosynthesis and carbon metabolism, Pearson correlation analysis was conducted across five growth stages. Pn was extremely significantly positively correlated with chlorophyll contents, soluble sugar and sucrose-metabolizing enzyme activities. Leaf iron and phosphorus contents were closely related to chlorophyll synthesis and enzyme activities respectively, with strongest correlations at fruit-setting, color-turning and maturity stages. These findings confirm the nutrient-photosynthesis-sucrose metabolism regulatory mechanism underlying improved leaf photosynthesis by nZV–KH_2_PO_4_ (Figure S2A–E).

### 2.2. Effects of nZVI and KH_2_PO_4_ on Fruit Sink Quality

#### 2.2.1. Fruit Appearance and Physical Traits

**Figure 3:**
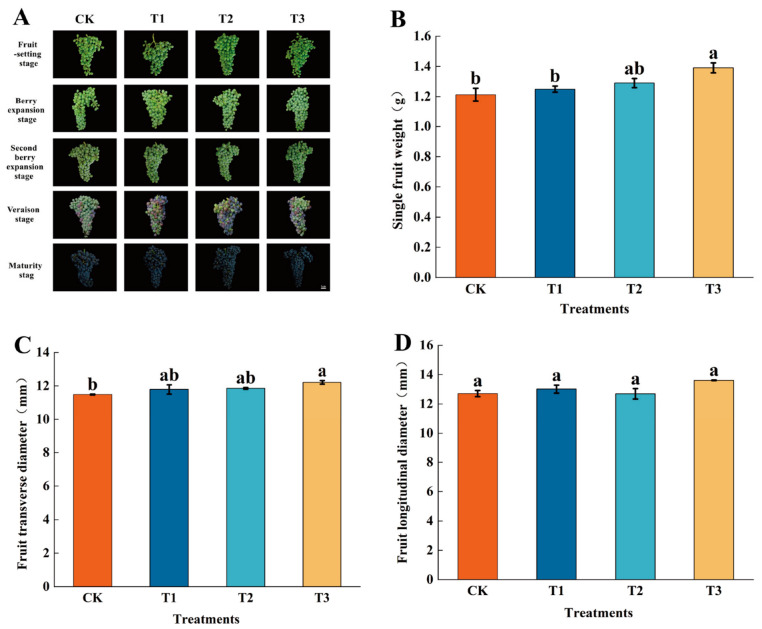
Foliar Application of Nanoscale Zero-Valent Iron, Potassium Dihydrogen Phosphate, and Their Combination Regulates Berry Phenotype and Fruit Growth Characteristics of Marselan Grape (*Vitis vinifera* L. cv. Marselan) Throughout the Whole Growth Cycle. (**A**) Phenotypic characteristics of grape clusters at five critical growth stages (fruit-setting stage, berry expansion stage, second berry expansion stage, veraison stage, and maturity stage) under different treatments. (**B**) Single berry weight of mature grapes (unit: g). (**C**) Transverse diameter of mature grape berries (unit: mm). (**D**) Longitudinal diameter of mature grape berries (unit: mm). Note: Different lowercase letters in the bar chart indicate significant differences (*p* < 0.05, one-way ANOVA, Tukey’s multiple comparison test) among treatments within the same parameter; identical letters indicate no significant difference. In the figure, orange, blue, light blue, and yellow correspond to the CK, T1, T2, and T3 treatments, respectively. Data are presented as mean ± standard error (n = 3). The same below.

Reinforced source function was effectively translated into improved fruit development and superior appearance quality (Figure 3A–D). Berries from T3-treated vines displayed more uniform growth dynamics and more synchronized coloration at maturity, with no visible underdeveloped or green fruits observed at harvest—a commercially important characteristic for premium grape production. Quantitative analysis demonstrated that single berry weight increased by 15.0% and transverse diameter by 6.1% relative to the control, indicating meaningful enhancement in fruit enlargement and final berry size. These improvements in physical traits are directly relevant to commercial grading standards and market acceptance, and underscore the practical agronomic value of the combined nano-fertilization strategy in high-value viticulture systems.

#### 2.2.2. Basic Quality Traits and Functional Components

**Figure 4:**
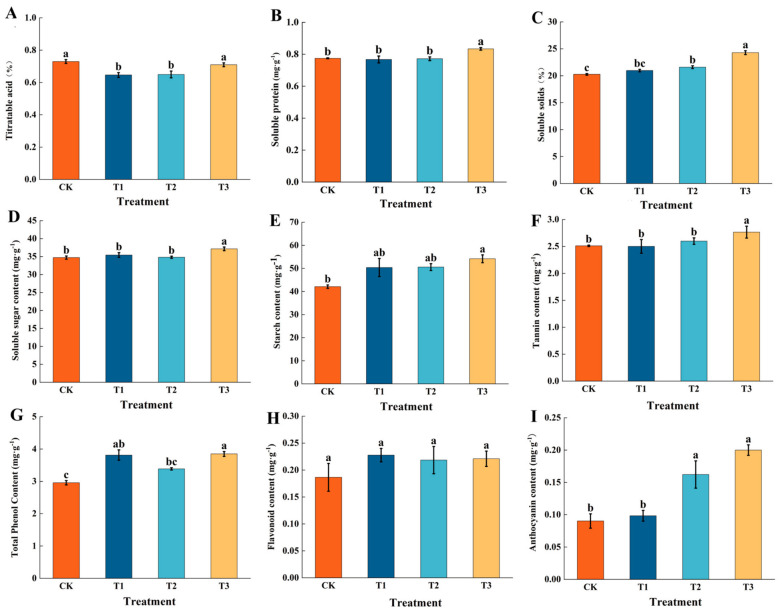
Foliar Application of Nanoscale Zero-Valent Iron, Potassium Dihydrogen Phosphate, and Their Combination Improves Nutritional Quality, Sugar Accumulation, and Functional Active Components of Mature Marselan Grape Berries. (**A**) Titratable acid content of mature grape berries (unit: %). (**B**) Soluble protein content of mature grape berries (unit: mg·g^−1^). (**C**) Soluble solids content of mature grape berries (unit: %). (**D**) Soluble sugar content of mature grape berries (unit: mg·g^−1^). (**E**) Starch content of mature grape berries (unit: mg·g^−1^). (**F**) Tannin content of mature grape berries (unit: mg·g^−1^). (**G**) Total phenol content of mature grape berries (unit: mg·g^−1^). (**H**) Flavonoid content of mature grape berries (unit: mg·g^−1^). (**I**) Anthocyanin content of mature grape berries (unit: mg·g^−1^). Note: Different lowercase letters in the bar chart indicate significant differences (*p* < 0.05, one-way ANOVA, Tukey’s multiple comparison test) among treatments within the same parameter; identical letters indicate no significant difference. In the figure, orange, blue, light blue, and yellow correspond to the CK, T1, T2, and T3 treatments, respectively. Data are presented as mean ± standard error (n = 3). The same below.

The combined treatment produced significant and coordinated improvements across key quality traits and functional components with direct relevance to industrial utilization (Figure 4A–I). Total soluble solids increased by 12.6%, accompanied by substantial gains in soluble sugars and starch, collectively reflecting enhanced berry sweetness and improved fermentable substrate availability for winemaking. Of particular industrial significance, bioactive compounds showed marked enrichment under T3. Total phenolics, condensed tannins, and anthocyanins were all significantly elevated, with anthocyanin content recording a 133.3% increase relative to the control—a finding of considerable importance given the critical role of anthocyanins in determining wine color stability, astringency modulation, and antioxidant capacity. These compounds are widely valued as functional ingredients in the food, nutraceutical, and cosmetic industries. The synergistic co-enhancement of basic quality parameters and high-value functional metabolites confirms that the combined nZVI/KH_2_PO_4_ treatment represents a uniquely effective strategy for simultaneously maximizing the nutritional and industrial value of grape berries.

#### 2.2.3. Analysis of Effects of nZVI and KH_2_PO_4_ on Fruit Organic Acids and Sugar

The combined treatment also produced a favorable reconfiguration of the sugar–acid balance (Figure S3A–H). Ascorbic acid content increased by 31.6%, contributing meaningfully to the enhanced total antioxidant potential of the berries. Regarding organic acid composition, non-characteristic acids—including oxalic, citric, and malic acids—were significantly reduced, while tartaric acid content remained stable, thereby preserving the characteristic acidic flavor profile typical of high-quality *Vitis vinifera* cultivars. Simultaneously, fructose, glucose, and sucrose contents were all significantly elevated, reflecting improved phloem-mediated assimilate transport and berry sink strength. This coordinated regulation of sugar accumulation and organic acid catabolism is central to achieving an optimal sugar–acid ratio, which governs perceived flavor balance, fermentation kinetics, and the suitability of the fruit for high-quality winemaking and functional food processing applications.

#### 2.2.4. Effects on Fruit Aroma Composition

Comprehensive volatile profiling identified 42 aroma-related compounds, with overall volatile abundance significantly greater under the combined treatment than in controls (Figure S4A–D). Key aroma-active compound classes—including C6 alcohols, monoterpenes, and aliphatic esters—were markedly enriched under T3, collectively enhancing the fruity, floral, and fresh aromatic character of the berries. Conversely, the abundance of undesirable aldehydes associated with green, herbaceous, or astringent off-notes was significantly reduced. These compositional shifts resulted in a more refined and balanced aroma profile, with improved sensory complexity. From an industrial standpoint, the selective enrichment of desirable volatile compounds directly elevates the aromatic complexity and market premium of grape-derived products—particularly in the context of premium wine production—where volatile composition is a key determinant of product differentiation and consumer preference.

### 2.3. Principal Component Analysis of Overall Fruit Quality

Principal component analysis (PCA) integrating 22 quality indicators demonstrated that the combined treatment (T3) achieved the highest comprehensive quality score among all experimental treatments (Table 2). The first three principal components collectively accounted for 100% of total variance, reflecting the high explanatory power of the selected quality descriptors and the robustness of the multivariate analysis framework. The treatment ranking (T3 > T2 > T1 > CK) unambiguously confirms that the combined application of nZVI and KH_2_PO_4_ delivers superior and comprehensive improvement across all dimensions of grape quality relative to either input applied alone. This integrated quality enhancement further substantiates the agronomic and commercial viability of this nano-fertilization strategy for sustainable premium viticulture.

### 2.4. Transcriptomic Responses to Combined nZVI and KH_2_PO_4_ Treatment

#### 2.4.1. Sequencing Quality Assessment

Following stringent quality filtering, high-quality clean reads were successfully obtained across all biological samples (Table 3). Sequencing quality metrics were uniformly robust: Q20 and Q30 values reached ≥94.43% and ≥94.27%, respectively, and GC content ranged from 46.97% to 47.24%, collectively confirming high base-call accuracy and sequence integrity. Clean reads were subsequently aligned to the *V. vinifera* reference genome (12X.v2), yielding overall mapping rates of ≥84.24%, unique mapping rates of ≥87.03%, and multi-mapped read fractions of ≤3.50% (Table 4). These quality parameters collectively demonstrate the reliability and statistical robustness of the transcriptomic dataset, providing a sound basis for downstream differential expression analysis and functional interpretation.

#### 2.4.2. Identification of Differentially Expressed Genes and Functional Enrichment

**Figure 5:**
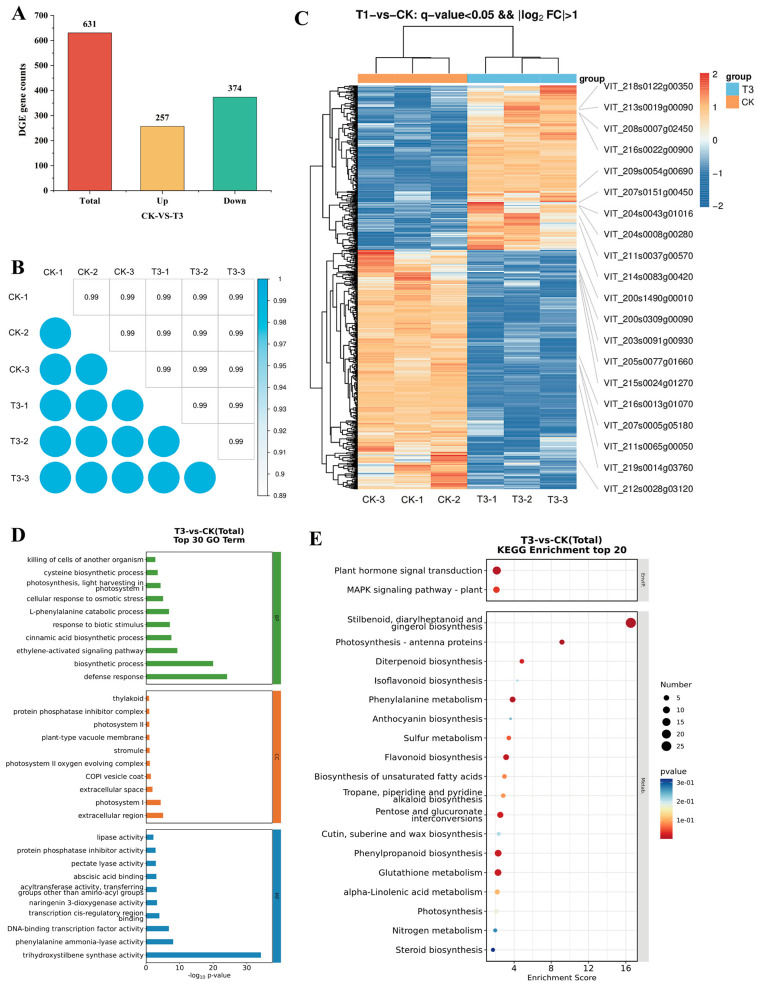
Transcriptomic Profiling Reveals Differential Gene Expression and Functional Pathway Enrichment in berry pericarp of Marselan grape berries in Response to Foliar Application of Nanoscale Zero-Valent Iron Alone and in Combination with Potassium Dihydrogen Phosphate. (**A**) Statistical analysis of DEGs between T3 treatment and CK control, based on grape leaves RNA colour change period (Stage IV). A total of 631 DEGs were identified, including 257 upregulated genes and 374 downregulated genes. (**B**) Pearson correlation coefficient analysis among all sequenced samples, including three biological replicates of CK (CK-1, CK-2, CK-3) and three biological replicates of T3 (T3-1, T3-2, T3-3). The size and color of the circles represent the value of the correlation coefficient. (**C**) Hierarchical clustering heatmap of DEGs between T3 treatment and CK control. The color scale represents the normalized expression level of genes, with red indicating upregulated expression and blue indicating downregulated expression. (**D**) Top 30 substantially enriched Gene Ontology (GO) terms of DEGs between T3 treatment and CK control, divided into three ontologies: biological process (BP, green), cellular component (CC, orange), and molecular function (MF, blue). (**E**) Top 20 markedly enriched KEGG pathways of DEGs between T3 treatment and CK control. The horizontal axis represents the enrichment score, the size of the dots represents the number of DEGs enriched in the corresponding pathway, and the color of the dots represents the *p*-value of the enrichment significance.

Differential expression analysis identified 631 differentially expressed genes (DEGs) under the combined treatment, signifying extensive and coordinated transcriptional reprogramming in response to nZVI/KH_2_PO_4_ co-application (Figure 5A–E). Gene Ontology (GO) and Kyoto Encyclopedia of Genes and Genomes (KEGG) enrichment analyses revealed that these DEGs were predominantly associated with photosynthesis, carbohydrate metabolism, and secondary metabolite biosynthesis—with particular enrichment in the phenylpropanoid and flavonoid biosynthesis pathways. These pathways are directly responsible for the synthesis of phenolics, anthocyanins, and other bioactive antioxidants, indicating that the combined treatment enhances grape functional quality not only at the physiological level but through targeted transcriptional activation of biosynthetic programs governing high-value metabolite production.

### 2.5. Metabolomic Responses to Combined Treatment

**Figure 6:**
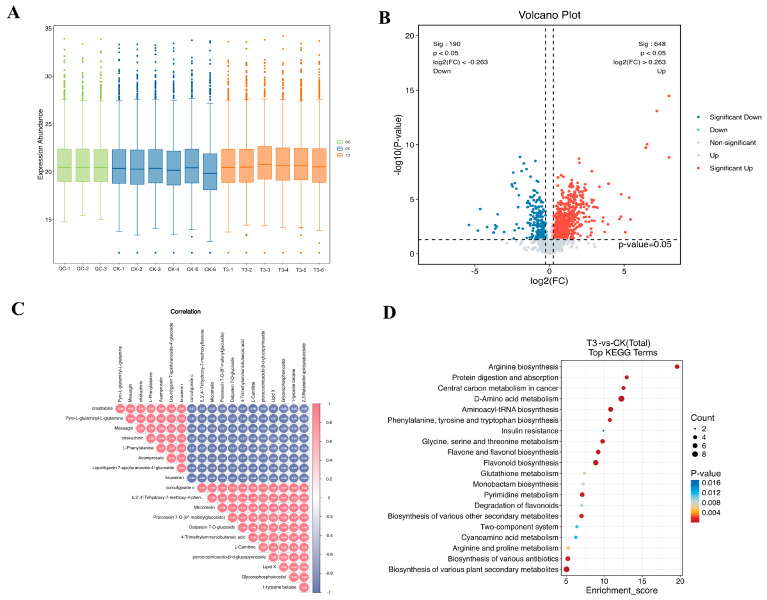
Metabolomic analysis of grape berry pericarp tissue at maturity (Stage V) reveals metabolite profile alterations and functional pathway enrichment in *V. vinifera* cv. Marselan treated with the combined application of nZVI and KH_2_PO_4_. (**A**) Box plot showing the distribution of metabolite expression abundance in QC samples, CK control samples (CK-1 to CK-6), and T3 treatment samples (T3-1 to T3-6), for evaluating the stability and repeatability of the metabolomic detection system. (**B**) Volcano plot of DAMs between T3 treatment and CK control. A total of 648 markedly upregulated metabolites and 190 significantly downregulated metabolites were identified. (**C**) Pearson correlation coefficient heatmap of key DAMs. The size and color of the circles represent the correlation coefficient between metabolites, with red indicating a positive correlation and blue indicating a negative correlation. (**D**) Top 20 considerably enriched KEGG pathways of DAMs between T3 treatment and CK control. The horizontal axis represents the enrichment score, the size of the dots represents the number of DAMs enriched in the corresponding pathway, and the color of the dots represents the *p*-value of the enrichment significance.

To complement the transcriptomic findings, untargeted metabolomic profiling was performed on grape berry pericarp tissue collected at the maturity stage (Stage V), consistent with the tissue source and sampling time point used for transcriptomic analysis. A total of 838 differentially accumulated metabolites (DAMs) were identified under T3 relative to controls, with a strong predominance of upregulated compounds (77.3%), indicating broad and preferentially positive metabolic reprogramming (Figure 6A–D). Network correlation analysis revealed strong positive associations among flavonoids, anthocyanins, and soluble sugars, consistent with a model of coordinated and mutually reinforcing metabolic regulation. These metabolites are recognized as primary determinants of berry antioxidant activity and industrial quality, confirming at the metabolome level that the combined treatment promotes the preferential accumulation of high-value functional compounds in a coherent and biologically integrated manner.

### 2.6. Integrated Transcriptomic and Metabolomic Analysis

#### 2.6.1. Core Regulatory Pathways

**Figure 7:**
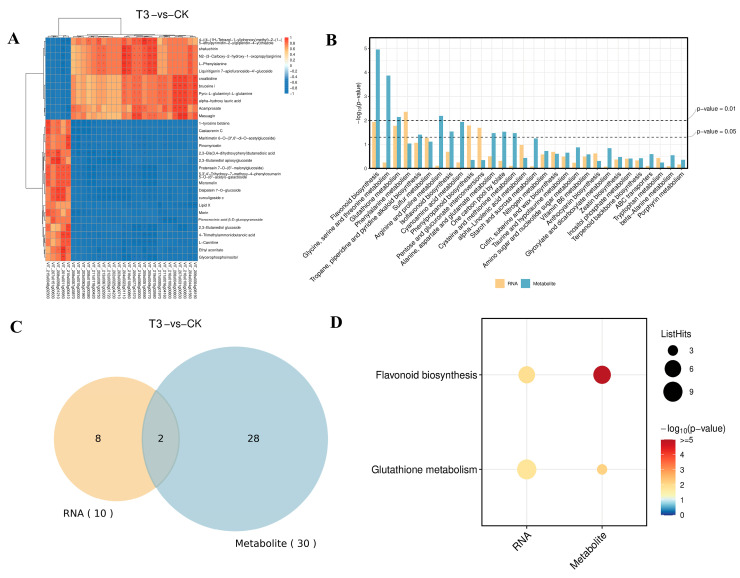
Integrated transcriptomic and metabolomic analysis reveals core regulatory pathways in Marselan grape berries in response to combined application of nanoscale zero-valent iron and dipotassium hydrogen phosphate. (**A**) Hierarchical clustering heatmap of co-expressed DEGs and DAMs between T3 treatment and CK control. The color scale represents the normalized expression level, with red indicating upregulated expression and blue indicating downregulated expression, *, **, and *** denote significance at *p* < 0.05, *p* < 0.01, and *p* < 0.001, respectively. (**B**) Bar chart of the considerably enriched Kyoto Encyclopedia of Genes and Genomes (KEGG) pathways shared by transcriptomic (RNA, yellow) and metabolomic (Metabolite, blue) datasets. The vertical axis represents the −log_10_ (*p*-value) of enrichment significance, with dashed lines marking the significance thresholds of *p* = 0.01 and *p* = 0.05. (**C**) Venn diagram showing the number of KEGG pathways uniquely and commonly enriched by DEGs (RNA) and DAMs (Metabolite) between T3 treatment and CK control. A total of 2 core pathways were co-enriched at both transcriptional and metabolic levels. (**D**) Scatter plot of core co-enriched KEGG pathways from the integrated transcriptomic and metabolomic analysis. The horizontal axis represents the enrichment level at the transcriptomic (RNA) level, and the vertical axis represents the enrichment level at the metabolomic level. The size of the dots indicates the number of enriched hits (genes/metabolites), and the color of the dots indicates the −log_10_ (*p*-value) of enrichment significance.

To comprehensively elucidate the coordinated molecular mechanisms underlying the observed physiological, phenotypic, and metabolic improvements, a joint analysis of DEGs and DAMs was conducted using a multi-omics integration framework. A total of 28 KEGG pathways were co-enriched at both the transcriptomic and metabolomic levels, among which flavonoid biosynthesis and glutathione metabolism emerged as the two most significantly enriched pathways (Figure 7). Bubble plot visualization revealed that flavonoid biosynthesis and glutathione metabolism exhibited exceptionally high enrichment factors of 16.2 and 12.8, respectively, with strong statistical significance (*p* < 0.01) (Figure 7B). Hierarchical heatmap clustering demonstrated consistent and concordant upregulation of both regulatory genes and their corresponding metabolite products within these pathways under T3 (Figure 7A). Quadrant plot analysis further confirmed that the vast majority of genes and metabolites associated with flavonoid biosynthesis and glutathione metabolism were simultaneously upregulated (Figure 7D), providing compelling evidence for tight transcription–metabolism coupling and coordinated regulatory co-activation under the combined treatment.

#### 2.6.2. Gene–Metabolite Regulatory Networks

**Figure 8:**
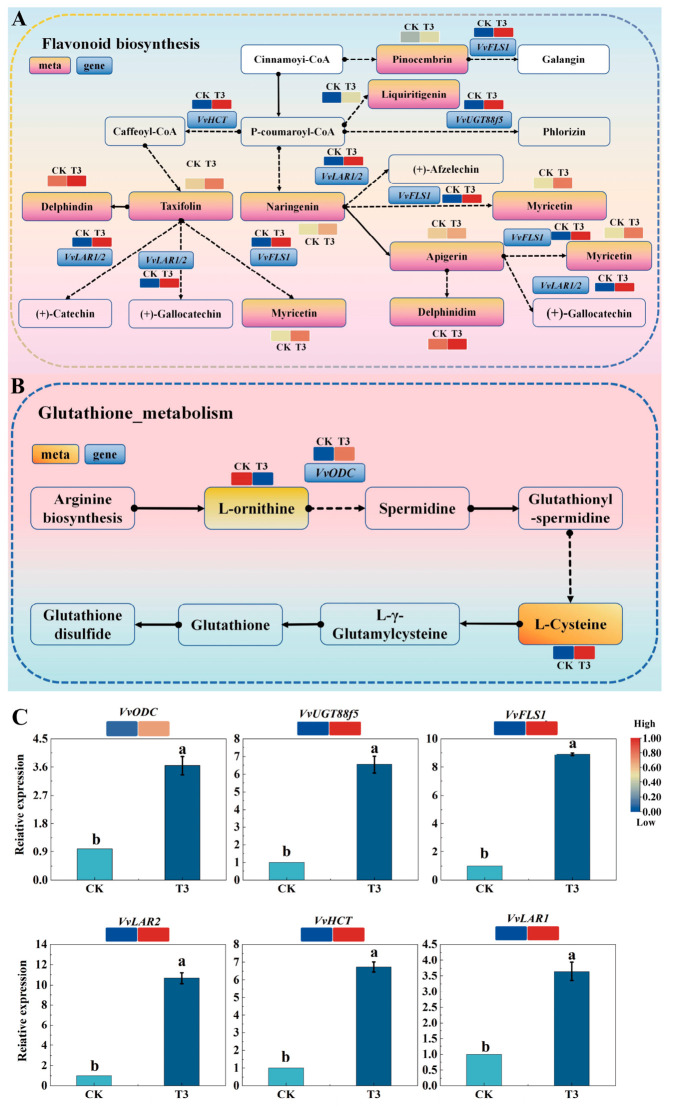
Integrated transcriptomic and metabolomic analysis combined with RT-qPCR validation reveals the regulatory effects of combined application of nanoscale zero-valent iron and potassium dihydrogen phosphate on key metabolic pathways and differentially expressed genes in Marselan grape berries. (**A**) Schematic diagram of the flavonoid biosynthesis pathway, showing the expression changes in key structural genes and metabolites between CK and T3 treatment. (**B**) Schematic diagram of the glutathione metabolism pathway, showing the expression changes in key structural genes and metabolites between CK and T3 treatment. (**C**) Relative expression levels of *VvODC*, *VvUGT88f5*, *VvFLS1*, *VvLAR2*, *VvHCT*, and *VvLAR1* genes. Explanation of gene abbreviations: *VvODC*, ornithine decarboxylase gene; *VvUGT88f5*, UDP-glycosyltransferase 88F5 gene; *VvFLS1*, flavonol synthase 1 gene; *VvLAR2*, leucoanthocyanidin reductase 2 gene; *VvHCT*, hydroxycinnamoyl transferase gene; *VvLAR1*, leucoanthocyanidin reductase 1 gene. The color blocks above each bar chart represent the expression levels of the corresponding genes derived from transcriptomic sequencing data, with the color scale ranging from blue (low expression) to red (high expression). Different lowercase letters above the bars indicate significant differences between the CK group and the T3 treatment group at *p* < 0.05 according to Duncan’s multiple range test.

Within the flavonoid and phenylpropanoid pathways (Figure 8A), the upstream gene *VvHCT* was significantly upregulated under T1 (~10.5-fold) and strongly positively correlated with L-phenylalanine and liquiritigenin 7-apiofuranoside-4′-glucoside (both R = 1.00, *p* < 0.001). *VvFLS1* (~5.7-fold) showed negative correlations with pinomyricetin, morin, and maritimetin 6-O-acetylglucoside (all R = −0.94, *p* = 0.005). *VvLAR1/2* and *VvUGT88F5* were also upregulated and correlated with key flavonoid metabolites (R = 0.83–1.00, *p* < 0.001). These changes align with activation of the MBW regulatory complex. In the glutathione pathway (Figure 8B), *VvODC* was elevated ~3.6-fold and positively correlated with L-ornithine, spermidine, and 1-tyrosine betaine (R = 0.82–0.94, *p* < 0.001 or *p* = 0.005), while negatively correlated with glutathione disulfide (R = −0.77, *p* < 0.01). This enhances redox homeostasis and antioxidant capacity, helping maintain berry quality under combined treatment (Figure 8C).

## 3. Discussion

Leaves constitute the primary photosynthetic organs of grapevines and function as the dominant source of photoassimilates underpinning berry development and quality formation. Their anatomical architecture, chloroplast structural integrity, and overall photosynthetic performance jointly govern carbon assimilation capacity and the efficiency of subsequent assimilate partitioning to reproductive sink organs. In the present study, the combined foliar application of nZVI and KH_2_PO_4_ produced marked and coordinated enhancements in leaf source function, as evidenced by concurrent improvements in mesophyll anatomy, chloroplast ultrastructure, and photosynthetic gas exchange parameters. These observations are broadly consistent with previous reports in cereal crops demonstrating that nZVI supplementation can stimulate chlorophyll biosynthesis and elevate photosynthetic efficiency [27,28,29]. Specifically, the combined treatment increased palisade tissue thickness and the chlorophyll a/b ratio, reflecting enhanced mesophyll compactness and augmented light-harvesting capacity per unit leaf area. Furthermore, the superior preservation of chloroplast ultrastructure—particularly the more organized and closely appressed thylakoid membrane stacking—provides a structural foundation for sustained photochemical activity and efficient linear electron flow [30]. These anatomical and ultrastructural adaptations are consistent with a model in which improved mineral nutrition reinforces the physical architecture of the photosynthetic apparatus, thereby potentiating its functional output under field conditions.

At the mechanistic level, the synergistic improvement in photosynthetic performance following combined nZVI and KH_2_PO_4_ foliar application stemmed from the complementary and mutually supportive regulatory effects of the two materials on nutrient homeostasis and photosynthetic metabolism in grapevine leaves. Vineyards are typically cultivated on alkaline calcareous soils, a prevalent abiotic stressor that severely constrains iron bioavailability and adversely affects grapevine vegetative growth and fruit development. Under high-pH soil conditions, conventional iron fertilizers rapidly undergo chemical precipitation and oxidation to form insoluble ferric (Fe^3+^) oxides and hydroxides, which are unavailable for root uptake; this process frequently induces leaf iron chlorosis, impairs chlorophyll biosynthesis and photosynthetic electron transport, and ultimately weakens leaf source capacity and restricts the accumulation of quality-related berry metabolites. Unlike traditional iron formulations, nZVI exhibits distinctive nanoscale physicochemical properties that effectively alleviate iron limitation under alkaline conditions, laying a critical structural and nutritional foundation for sustained photosynthetic function. Benefiting from its high specific surface area, strong surface adsorption capacity, and controlled nutrient release characteristics, nZVI effectively counteracts high-pH-induced iron precipitation and inactivation. Foliar-sprayed nZVI can stably attach to the leaf epidermis and stomatal apparatus, penetrate the leaf cuticle and cell wall via its nanosize effect, and continuously release bioavailable ferrous iron (Fe^2+^) within leaf mesophyll tissues and the apoplastic microenvironment, thereby preventing rapid iron oxidation and nutrient failure under alkaline physiological conditions. Additionally, nZVI moderately modulates the local micro-pH surrounding leaf mesophyll cells, mitigates microenvironmental alkalinity, and further suppresses iron oxidation and sedimentation, consequently maintaining a stable and long-term supply of active Fe^2+^ in leaf tissues. This sustained iron bioavailability substantially alleviates alkaline-soil-induced iron deficiency stress in grapevines. The sufficient iron supply further promotes the biosynthesis of chlorophyll precursors, particularly protochlorophyllide formation, and stabilizes chlorophyll–protein complexes within the PSII reaction center. These protective effects maintain the photochemical quantum efficiency (*Fv*/*Fm*) and alleviate high-light-induced photoinhibition, consistent with the essential role of iron as a key cofactor in the photosynthetic electron transport chain and oxygen-evolving complex. In parallel, KH_2_PO_4_ supplies readily absorbable phosphorus, an essential macronutrient required for the synthesis of ATP, NADPH, and membrane phospholipids. Adequate phosphorus nutrition enhances the activities of key sucrose metabolism enzymes, including sucrose phosphate synthase (SPS) and sucrose synthase (SS), thereby accelerating sucrose synthesis, phloem loading, and the accumulation of soluble sugars and transient starch [31]. Furthermore, sufficient phosphorus optimizes cellular ATP/ADP and NADPH/NADP^+^ ratios to facilitate the Calvin–Benson cycle, improves Rubisco carboxylation efficiency and triose phosphate export, and consequently elevates net carbon fixation capacity [31]. Foliar phosphorus application also facilitates stomatal opening via guard cell osmoregulation, further promoting photosynthetic carbon assimilation under favorable light conditions [32].

These complementary structural and physiological improvements collectively establish an integrated and self-reinforcing regulatory framework that can be conceptualized as: photosynthetic structure protection → photochemical efficiency enhancement → carbon metabolic activation → assimilate accumulation. This cascade ultimately strengthens leaf source capacity and ensures a sustained and sufficient carbon supply to support fruit development and the biosynthesis of quality-determining metabolites. According to source–sink theory, enhanced source strength is a primary determinant of sink organ development and quality formation [33]. Consistent with this framework, the improved leaf performance observed under T3 was accompanied by significant and coordinated enhancements in fruit quality traits, including increased single berry weight, improved size uniformity, elevated total soluble solids, and a more favorable sugar–acid ratio. Notably, the stability of tartaric acid—the characteristic organic acid of *Vitis vinifera* berries—indicates preservation of cultivar-specific flavor typicity, while the moderate reduction in malic and citric acid concentrations contributes to attenuation of excessive acidity and improves palatability [34,35,36]. These outcomes align with the enological requirements for premium wine production, in which both sufficient sugar accumulation and appropriate acid balance are critical determinants of final product quality.

Metabolomic profiling further demonstrated that the combined treatment markedly promoted the accumulation of the major soluble sugars—fructose, glucose, and sucrose—which are the primary determinants of berry sweetness and the foundation of the sugar–acid equilibrium. The expanded availability of carbon skeletons also fueled secondary metabolic flux, particularly through the phenylpropanoid–flavonoid biosynthetic axis, resulting in significantly elevated levels of total phenolics, flavonoids, anthocyanins, and condensed tannins. These bioactive compounds are recognized as key contributors to the nutritional functionality, color stability, and sensory complexity of wine grapes and their derived products [37,38]. Correlation network analysis revealed a tightly coordinated metabolic architecture linking sugars, flavonoids, and anthocyanins, indicating that carbon flux redistribution—driven by enhanced photosynthetic source output—constitutes a central mechanistic determinant of the observed quality improvements. These results support a mechanistic framework of: enhanced carbon supply → secondary metabolic pathway activation → coordinated accumulation of quality-determining metabolites, underscoring the primacy of source-driven carbon allocation in regulating berry functional quality.

Integrated transcriptomic and metabolomic analysis provided deeper mechanistic resolution into the molecular basis of these coordinated responses. Flavonoid biosynthesis and glutathione metabolism emerged as the two most significantly co-enriched KEGG pathways, highlighting their central and indispensable roles in quality-related metabolic regulation. Within the phenylpropanoid–flavonoid pathway, the marked upregulation of the hydroxycinnamoyl transferase gene *VvHCT* under T3 is predicted to enhance the production of pivotal pathway intermediates—including caffeoyl-CoA and p-coumaroyl-CoA—thereby expanding the availability of substrates for downstream flavonoid biosynthesis [39]. Concurrently, the coordinated upregulation of core structural genes—*VvFLS1*, *VvLAR1*, *VvLAR2*, and *VvUGT88F5*—with expression increases of up to 8–10-fold, was strongly and significantly associated (*p* < 0.001) with the enhanced accumulation of flavonoids and anthocyanins, including naringenin, kaempferol, quercetin, catechin, and delphinidin [40]. Given the well-characterized roles of FLS in flavonol biosynthesis, LAR in proanthocyanidin (condensed tannin) formation, and HCT as a critical regulatory node linking the general phenylpropanoid pathway to the flavonoid branch, their coordinated transcriptional activation represents a key molecular mechanism underlying the nZVI/KH_2_PO_4_-driven enhancement of flavonoid accumulation [41,42]. It is plausible that these transcriptional responses could be partly mediated by nZVI-induced reactive oxygen species (ROS) signaling at sub-toxic concentrations, which has been shown to activate MYB-type transcription factors governing flavonoid pathway gene expression in grapevine; however, this hypothesis remains untested in the absence of direct ROS measurements or redox status analysis in the present study. Recent studies support this framework: a 2025 study demonstrated that foliar application of nZVI significantly enhanced leaf photosynthetic rate, antioxidant enzyme activities, and fruit quality in apple [43], and a 2025 transcriptomic study identified *MYB24* as a key coordinator of flavonol biosynthesis in grape [44].

In addition to secondary metabolic reprogramming, glutathione metabolism emerged as an equally important regulatory axis in the integrated multi-omics network. It is important to note that the link between *VvODC* upregulation and enhanced glutathione accumulation is indirect and speculative. The elevated expression of *VvODC*—encoding ornithine decarboxylase—suggests enhanced polyamine biosynthetic flux, and might theoretically contribute to glutathione production indirectly via increased availability of ornithine-derived precursor molecules or shared pathway intermediates [45,46]; however, this proposed connection remains hypothetical and requires direct experimental validation. As a central hub of non-enzymatic antioxidant defense, the glutathione redox system plays indispensable roles in scavenging reactive oxygen species, detoxifying lipid peroxidation products, and maintaining cellular redox homeostasis [47,48]. The consequent enhancement of antioxidant capacity is expected to mitigate oxidative damage to photosynthetic membranes, attenuate chlorophyll degradation, and delay both leaf and fruit senescence—ultimately sustaining metabolic activity throughout the critical window of berry ripening. This regulatory mechanism can be conceptualized as: enhanced antioxidant capacity → oxidative stress mitigation → senescence delay → sustained metabolic and photosynthetic function, which provides an additional molecular rationale for the superior and prolonged performance of the combined treatment observed at both the physiological and metabolic levels.

From an applied perspective, foliar application of 15 mg·L^−1^ nZVI combined with 1.67 g·L^−1^ KH_2_PO_4_ proved effective in comprehensively improving grape berry quality across multiple dimensions, while remaining operationally straightforward, economically accessible, and environmentally compatible with the principles of sustainable agriculture. These attributes collectively suggest strong potential for practical adoption and scalable implementation in commercial viticulture systems. Nevertheless, several limitations of the present study merit explicit acknowledgment. The transcriptomic and metabolomic analyses were conducted at a single developmental time point (ripening stage), which constrains our capacity to fully resolve the temporal dynamics of gene expression and metabolite accumulation across the entire berry developmental trajectory. Additionally, while the key regulatory genes identified herein—*VvHCT*, *VvFLS1*, *VvLAR1/2*, *VvUGT88F5*, and *VvODC*—exhibited strong expression–accumulation correlations, their specific functional contributions remain to be experimentally validated through loss-of-function or gain-of-function approaches. Future investigations integrating spatiotemporally resolved multi-omics profiling across critical developmental windows—from fruit set through post-veraison ripening—combined with functional validation strategies such as CRISPR/Cas9-mediated gene editing or virus-induced gene silencing (VIGS), will be essential to fully elucidate the regulatory architecture governing nZVI/KH_2_PO_4_-mediated quality enhancement in grapevine [49,50,51]. Such mechanistic advances will further strengthen the scientific foundation for the rational design of nanomaterial-assisted precision fertilization strategies applicable to a broad range of high-value horticultural crops.

## 4. Materials and Methods

### 4.1. Plant Materials and Experimental Design

#### 4.1.1. Plant Materials

The experiment was conducted from June to October 2024 at Yinuo Winery, Wuwei, Gansu Province, China (102°82′26″ E, 37°18′06″ N). Five-year-old *Vitis vinifera* cv. Marselan vines were used as experimental materials. Vines were planted in an east–west orientation with a spacing of 3.5 m between rows and 1.0 m between plants and trained using a single-arm trellis system. Standard vineyard management practices were uniformly applied across all treatments. The soil in the experimental site was classified as neutral to slightly alkaline sandy loam. The region has a typical temperate arid climate, with an annual precipitation of 262.9 mm, annual sunshine duration of 2876.9 h, and a mean annual temperature of 7.1 °C. During the experimental period (summer season), the diurnal temperature variation was approximately 15 °C, and the hydrothermal coefficient remained below 1.5.

#### 4.1.2. Experimental Design

According to previous research [25], four treatments were established: water control (CK), nZVI was provided by Gansu Gushuo Nano Agricultural Science and Technology Co., Ltd. KH_2_PO_4_ was provided by Sichuan Guoguang Agrochemical Co., Ltd. and their combined application. nZVI was provided by Gansu Gushuo Nano Agricultural Science and Technology Co., Ltd. (Lanzhou, China). KH_2_PO_4_ was provided by Sichuan Guoguang Agrochemical Co., Ltd. (Chengdu, China). Details of foliar fertilization treatments are provided in Table 5. Healthy vines with uniform growth were selected to ensure consistent initial conditions. Each treatment included three biological replicates, with five vines per replicate (total of 45 vines). Foliar spraying was conducted at five phenological stages: fruit set, berry enlargement, secondary enlargement, veraison, and ripening. Applications were performed at approximately 9:00 a.m. on clear days until leaf surfaces were fully wetted (dripping point), with a spray volume of 2 L per vine. The control treatment received an equivalent volume of water.

#### 4.1.3. Sampling

At each phenological stage, ten functional leaves per treatment were randomly collected 10 d after spraying. A portion of the samples was immediately fixed in FAA solution for anatomical analysis, while the remaining samples were frozen in liquid nitrogen and stored at −80 °C for physiological measurements. At the ripening stage, healthy berries from the portion of clusters were collected, immediately frozen in liquid nitrogen, and stored at −80 °C for fruit quality analysis and omics sequencing.

### 4.2. Measurements and Analytical Methods

#### 4.2.1. Leaf Anatomical and Ultrastructural Analysis

Leaf anatomical structure was analyzed using the paraffin sectioning method. FAA-fixed samples were dehydrated, cleared, embedded in paraffin, and sectioned at 8 μm thickness. Sections were stained with safranin–fast green and observed under a light microscope. Palisade tissue thickness, spongy tissue thickness, and total leaf thickness were measured using ImageJ software (version 1.54k, National Institutes of Health, Bethesda, MD, USA), and palisade tissue compactness (CTR%) and spongy tissue looseness (SR%) were calculated. Stomatal morphology was observed using scanning electron microscopy (SEM, Hitachi SU8010, Hitachi High-Technologies Corporation, Tokyo, Japan) after fixation in 2.5% glutaraldehyde, dehydration, critical-point drying, and gold coating. Chloroplast ultrastructure was examined using transmission electron microscopy (TEM, Hitachi HT7700, Hitachi High-Technologies Corporation, Tokyo, Japan) following standard sample preparation procedures.

#### 4.2.2. Photosynthetic Parameters

Chlorophyll a and b contents were determined using the acetone–ethanol extraction method. Net photosynthetic rate (*Pn*), stomatal conductance (*Gs*), intercellular CO_2_ concentration (*Ci*), and transpiration rate (*Tr*) were measured using a LI-6400XT portable photosynthesis system between 9:00 and 11:00 a.m. under a light intensity of 1200 μmol·m^−2^·s^−1^. Five functional leaves per replicate were measured. Chlorophyll fluorescence parameters, including minimal fluorescence (*F*_0_) and maximum quantum efficiency of PSII (*Fv*/*Fm*), were measured using a Junior-PAM fluorometer after 20 min of dark adaptation.

#### 4.2.3. Carbon Metabolism and Nutrient Analysis

Soluble protein content was determined using the Coomassie Brilliant Blue method. Total soluble sugars and starch were measured using the anthrone method. Fructose, glucose, and sucrose contents were quantified by high-performance liquid chromatography (HPLC). Activities of sucrose metabolism-related enzymes, including neutral invertase (NI), sucrose phosphate synthase (SPS), acid invertase (AI), and sucrose synthase (SS), were determined using enzyme-linked immunosorbent assay (ELISA) kits (Shanghai Enzyme-linked Biotechnology Co., Ltd., Shanghai, China). Leaf Fe and P concentrations were measured using inductively coupled plasma optical emission spectrometry (ICP-OES, iCAP 7400, Thermo Fisher Scientific Ltd., Cambridge, UK).

#### 4.2.4. Fruit Quality Analysis

Single berry weight was measured using an analytical balance (30 berries per replicate). Berry longitudinal and transverse diameters were measured using a digital caliper. Titratable acidity was determined by NaOH titration, and total soluble solids were measured using a handheld refractometer. Ascorbic acid and organic acids (oxalic, tartaric, citric, and malic acids) were quantified by HPLC. Total phenolic content was determined using the Folin–Ciocalteu method, total flavonoids were measured using the aluminum nitrate colorimetric method, tannin content was determined using the vanillin–HCl method, and anthocyanin content was measured using the pH differential method.

#### 4.2.5. Aroma Compound Analysis

Volatile compounds were analyzed using headspace solid-phase microextraction (HS-SPME, MPS XL, GERSTEL GmbH & Co. KG, Mülheim an der Ruhr, Germany) coupled with gas chromatography–mass spectrometry (GC-MS, 7890A-5975C, Agilent Technologies, Inc., Santa Clara, CA, USA). Briefly, 5 g of homogenized fruit sample was placed in a 20 mL headspace vial, followed by the addition of 1.5 g NaCl and 10 μL of 2-octanol as an internal standard. Samples were equilibrated at 55 °C for 10 min and extracted using a 50/30 μm DVB/CAR/PDMS fiber for 30 min. After thermal desorption (5 min), compounds were analyzed using GC–MS equipped with an HP-5MS capillary column. Identification was performed using the NIST14 mass spectral library, and quantification was conducted using the internal standard method.

### 4.3. Transcriptomic and Metabolomic Analyses

Flesh samples from CK and T3 treatments at the ripening stage (three biological replicates) were used for transcriptomic and metabolomic analyses. Total RNA was extracted using the TRIzol method, and RNA quality was assessed prior to cDNA library construction. Sequencing was performed on the Illumina NovaSeq 6000 platform. For metabolomic analysis, metabolites were extracted using 40% methanol–acetonitrile solution and analyzed using an ultra-performance liquid chromatography–tandem mass spectrometry (UPLC–MS/MS) platform for untargeted metabolomics.

### 4.4. Integrated Transcriptomic and Metabolomic Analysis

Differentially expressed genes (DEGs) and differentially accumulated metabolites (DAMs) were subjected to Kyoto Encyclopedia of Genes and Genomes (KEGG) pathway enrichment analysis to identify commonly enriched pathways. Pearson correlation analysis was performed to construct gene–metabolite interaction networks within key pathways.

### 4.5. qRT-PCR Validation

Six key genes involved in core pathways were selected for validation. Primers were designed using Primer 5.0 software (sequences listed in Table 6), and Actin was used as the internal reference gene. qRT-PCR was performed using the SYBR Green method under the following conditions: 95 °C for 30 s, 40 cycles of 95 °C for 5 s and 60 °C for 30 s. Relative gene expression levels were calculated using the 2^−ΔΔCt^ method with three technical replicates.

### 4.6. Statistical Analysis

Data were analyzed using SPSS 26.0 software. One-way analysis of variance (ANOVA) followed by Duncan’s multiple range test was used to determine significant differences at *p* < 0.05. Principal component analysis (PCA) was performed using IBM SPSS Statistics 26.0 (IBM, New York, NY, USA). Correla-tion heatmaps and figures were generated with Origin 2023 (OriginLab Corporation, Northampton, MA, USA).

## 5. Conclusions

**Figure 9:**
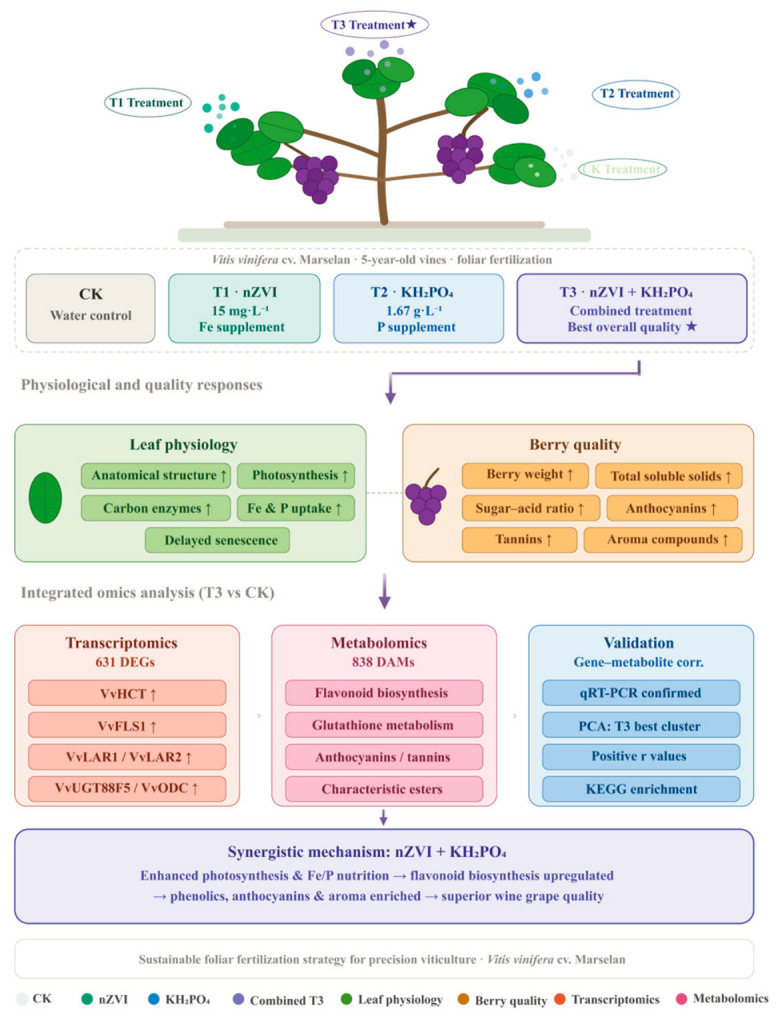
Synergistic Enhancement of Winemaking Quality in *Vitis vinifera* L. cv. Marselan Grape by Nano Zero-Valent Iron and Potassium Dihydrogen Phosphate: Physiological, Transcriptomic, and Metabolomic Mechanisms.

Foliar co-application of nZVI and KH_2_PO_4_ comprehensively enhances berry quality in *Vitis vinifera* cv. Marselan through coordinated source–sink regulation (Figure 9). At the source level, the combined treatment reinforced photosynthetic capacity, stimulated sucrose metabolism, and optimized Fe and P utilization, collectively amplifying carbon supply to developing berries. This strengthened source output was functionally coupled to preferential activation of flavonoid biosynthesis and glutathione metabolism, resulting in elevated phenolic and anthocyanin accumulation, improved sugar–acid balance, and enhanced antioxidant capacity. Integrated multi-omics analysis identified *VvHCT*, *VvFLS1*, *VvLAR1*/*2*, *VvUGT88F5*, and *VvODC* as central regulatory nodes linking transcriptional reprogramming to metabolite accumulation. These findings establish a practical and environmentally compatible nano-fertilization strategy for improving the functional and industrial value of premium wine grapes.

## Figures and Tables

**Table 1:** Effects of different treatments on leaf structure indexes of Marselan grapes.

Stage	Treatment	Palisade Tissue Thickness (μm)	Spongy Tissue Thickness (μm)	Leaf Thickness (μm)	CTR%	SR%
Fruit-setting stage	CK	80.00 ± 1.15 c	78.66 ± 10.17 a	198.00 ± 2.64 b	40.40	39.27
	T1	83.00 ± 4.25 b	84.30 ± 2.84 a	205.33 ± 4.25 ab	40.77	41.05
	T2	84.67 ± 2.02 a	84.67 ± 3.84 a	206.00 ± 1.52 ab	40.65	40.65
	T3	87.33 ± 1.45 a	85.00 ± 0.57 a	214.66 ± 2.40 a	40.68	39.59
Berry expansion stage	CK	83.66 ± 1.85 a	75.60 ± 1.45 a	218.00 ± 4.04 a	38.37	34.67
	T1	85.00 ± 0.57 a	76.67 ± 2.02 a	221.33 ± 6.48 a	38.40	34.64
	T2	85.00 ± 1.52 a	76.33 ± 4.48 a	220.67 ± 0.33 a	38.63	34.59
	T3	86.00 ± 2.64 a	77.30 ± 6.69 a	223.00 ± 6.80 a	38.56	34.66
Second berry expansion stage	CK	82.66 ± 0.33 a	72.00 ± 5.50 a	203.00 ± 1.73 a	40.71	35.46
	T1	84.00 ± 0.57 a	76.00 ± 3.05 a	206.00 ± 3.75 a	40.77	36.89
	T2	83.00 ± 2.08 a	75.66 ± 4.91 a	205.00 ± 2.64 a	40.48	36.90
	T3	81.67 ± 2.72 a	71.33 ± 1.85 a	199.00 ± 0.57 a	40.70	35.84
Veraison stage	CK	80.33 ± 0.88 bc	68.33 ± 1.20 a	198.00 ± 4.35 a	40.40	34.51
	T1	85.66 ± 1.20 a	72.66 ± 3.28 a	200.00 ± 5.50 a	42.83	36.33
	T2	78.67 ± 1.45 c	66.60 ± 6.11 a	188.66 ± 0.88 a	41.48	35.30
	T3	84.30 ± 0.88 ab	71.00 ± 0.57 a	195.00 ± 2.64 a	43.23	36.41
Maturity stage	CK	76.00 ± 1.52 c	63.31 ± 4.05 a	188.00 ± 4.93 a	40.42	33.67
	T1	83.60 ± 1.20 a	69.67 ± 2.02 a	191.00 ± 0.57 a	43.45	36.47
	T2	78.33 ± 1.20 bc	65.00 ± 5.50 a	190.67 ± 1.20 a	40.90	34.09
	T3	81.66 ± 0.88 ab	61.33 ± 0.88 a	189.00 ± 1.52 a	42.85	32.44

Note: Data in the table are presented as mean ± standard error (n = 3). Different lowercase letters (a, b, c) within the same column indicate significant differences among treatments at the same developmental stage (*p* < 0.05, one-way ANOVA, Tukey’s multiple comparison test); treatments marked with the same letter show no significant differences. The same below.

**Table 2:** Principal component scores table for the effects of different treatments on photosynthetic cCharacteristics and related nutritional indices in marselan grape leaves.

Treatment	FAC1	FAC2	FAC3	Comprehensive Score	Ranking
CK	−1.24	−0.64	−0.54	−0.93	4
T1	0.33	−0.59	1.34	0.24	3
T2	1.14	−0.24	−0.95	0.32	2
T3	−0.22	1.48	0.15	0.36	1
Eigenvalues	7.09	4.26	2.66		
Variance contribution rate/%	50.63	30.4	18.97		
Cumulative variance contribution rate/%	50.63	81.03	100		

**Table 3:** Sequencing data statistics.

Sample	Raw Reads	Clean Reads	Clean Base (G)	Error Rate (%)	Q20 (%)	Q30 (%)	GC Content (%)
CK-1	25.66	7.45	24.23	7.03	94.43	94.43	47.20
CK-2	25.21	7.34	23.94	6.97	94.96	94.50	47.19
CK-3	25.50	7.44	24.22	7.07	94.98	94.27	47.24
T3-1	25.15	7.33	23.92	6.97	95.12	94.56	47.06
T3-2	24.84	7.25	23.66	6.90	95.23	94.76	46.97
T3-3	25.21	7.33	23.81	6.92	94.45	94.29	46.98

**Table 4:** Comparison results with the reference genome.

Sample	Reads Mapped	Unique Mapped	Multi Mapped
CK-1	40,818,548 (84.24%)	42,173,739 (87.03%)	1,684,715 (3.48%)
CK-2	40,565,682 (84.74%)	41,917,647 (87.56%)	1,677,574 (3.50%)
CK-3	40,932,260 (84.49%)	42,322,487 (87.36%)	1,637,461 (3.38%)
T3-1	40,587,054 (84.84%)	41,899,640 (87.59%)	1,563,570 (3.27%)
T3-2	40,160,696 (84.88%)	41,454,067 (87.61%)	1,531,116 (3.24%)
T3-3	40,376,492 (84.78%)	41,454,067 (87.61%)	1,540,621 (3.24%)

**Table 5:** Foliar Spraying Foliar Fertilizer Programs.

Treatment	nZV (mg·L^−1^)	KH_2_PO_4_ (g·L^−1^)	Purity (%)
CK	0	0	-
T1	15	0	≥98 (BR)
T2	0	1.67	≥98 (BR)
T3	15	1.67	≥98 (BR)

**Table 6:** Primer sequence.

Primer Name	Primer Sequence (5′ → 3′)	Applications
*VvODC-F*	CGATGACGCCACTTCTGCTATC	qRT-PCR
*VvODC-R*	TCCGACCTCACACGCTTCC	qRT-PCR
*VvUGT88f5-FORWARD*	TCCGTCTCAGTGCCTCTAATGTC	qRT-PCR
*VvUGT88f5-REVERSE*	AATTCCAAGGTCACGAGCAACAG	qRT-PCR
*VvFLS1-FORWARD*	ACATCTCCAACGCTCTTGTCATTC	qRT-PCR
*VvFLS1-REVERSE*	CATCCTCGTCTTCTCCTTGTTCAC	qRT-PCR
*VvLAR2-FORWARD*	CCGTGTAACCGTGGAAGAAGATG	qRT-PCR
*VvLAR2-REVERSE*	ATCCCTTTATGAAGATGTCGTGAGTG	qRT-PCR
*VvHCT-FORWARD*	AGAGCGGAAGAGAGAAACCACTAC	qRT-PCR
*VvHCT-REVERSE*	GATGTGTCCAGCAATAGCCTCATAC	qRT-PCR
*VvLAR1-FORWARD*	AGAGTGTGGTAGCGGCGTTC	qRT-PCR
*VvLAR1-REVERSE*	AAGCATTCCTCCACCGTCCTG	qRT-PCR

## Data Availability

The data presented in this study are available on request from the corresponding author.

## Supplementary Materials

The following supporting information can be downloaded at: https://www.mdpi.com/article/10.3390/plants15111595/s1, Figure S1: Foliar Application of Nanoscale Zero-Valent Iron, Potassium Dihydrogen Phosphate, and Their Combination Modulates Carbohydrate Metabolism, Key Metabolic Enzyme Activities, and Mineral Element Accumulation in Marselan Grape Leaves Across the Whole Growth Cycle. (A) Soluble protein content in grape leaves (unit: mg·g^−1^). (B) Soluble sugar content in grape leaves (unit: mg·g^−1^). (C) Starch content in grape leaves (unit: mg·g^−1^). (D) Fructose content in grape leaves (unit: mg·g^−1^). (E) Glucose content in grape leaves (unit: mg·g^−1^). (F) Sucrose content in grape leaves (unit: mg·g^−1^). (G) Neutral invertase (NI) activity in grape leaves (unit: U·g^−1^ FW, FW: fresh weight). (H) Sucrose phosphate synthase (SPS) activity in grape leaves (unit: U·g^−1^ FW). (I) Iron content in grape leaves (unit: mg·kg^−1^). (J) Soluble acid invertase (SAI) activity in grape leaves (unit: U·g^−1^ FW). (K) Sucrose synthase (SS) activity in grape leaves (unit: U·g^−1^ FW). (L) Phosphorus content in grape leaves (unit: g·kg^−1^).; Figure S2: Pearson Correlation Analysis Between Leaf Photosynthetic Traits, Photosynthetic Pigment Contents, and Carbohydrate Metabolism Parameters of Marselan Grape (*Vitis vinifera* L. cv. Marselan) Across Five Key Growth Stages. Correlation heatmap at the fruit-setting stage. (B) Correlation heatmap at the berry expansion stage. (C) Correlation heatmap at the second berry expansion stage. (D) Correlation heatmap at the veraison stage. (E) Correlation heatmap at the maturity stage. Correlation analysis was conducted based on physiological indicators measured at five critical grape growth stages. Abbreviations for physiological indicators: Pn, net photosynthetic rate; Tr, leaf transpiration rate; Gs, leaf stomatal conductance; Ci, leaf intercellular carbon dioxide concentration; F_0_, initial fluorescence of Photosystem II (PSII); Fv/Fm, maximum photochemical efficiency of PSII. The color scale represents the value of the Pearson correlation coefficient, ranging from −1 (green, negative correlation) to 1 (red, positive correlation). Asterisks indicate significant correlation: * *p* < 0.05, ** *p* < 0.01, *** *p* < 0.001.; Figure S3: Oliar Application of Nanoscale Zero-Valent Iron, Potassium Dihydrogen Phosphate, and Their Combination Alters Organic Acid and Soluble Sugar Components in Mature Marselan Grape Berries. Ascorbic acid content in mature grape berries (unit: mg·g^−1^). (B) Oxalic acid content in mature grape berries (unit: mg·g^−1^). (C) Tartaric acid content in mature grape berries (unit: mg·g^−1^). (D) Citric acid content in mature grape berries (unit: mg·g^−1^). (E) Malic acid content in mature grape berries (unit: mg·g^−1^). (F) Fructose content in mature grape berries (unit: mg·g^−1^). (G) Glucose content in mature grape berries (unit: mg·g^−1^). (H) Sucrose content in mature grape berries (unit: mg·g^−1^).; Figure S4: Foliar Application of Nanoscale Zero-Valent Iron, Potassium Dihydrogen Phosphate, and Their Combination Modulates the Profile and Relative Content of Volatile Aroma Compounds in Mature Marselan Grape Berries. Volatile aroma compound profile of mature grape berries in the CK treatment. (B) Volatile aroma compound profile of mature grape berries in the T1 treatment. (C) Volatile aroma compound profile of mature grape berries in the T2 treatment. (D) Volatile aroma compound profile of mature grape berries in the T3 treatment.
