# Supplementary figures and images for: Synergistic Effects of nZVI and KH2PO4 on Phenolic Accumulation, Antioxidant Capacity and Fruit Quality of Marselan Grape via Multi-Omics
Source: Plants (Basel). 2026 May 22;15(11):1595. doi: 10.3390/plants15111595 (PMC13259039; doi:10.3390/plants15111595)

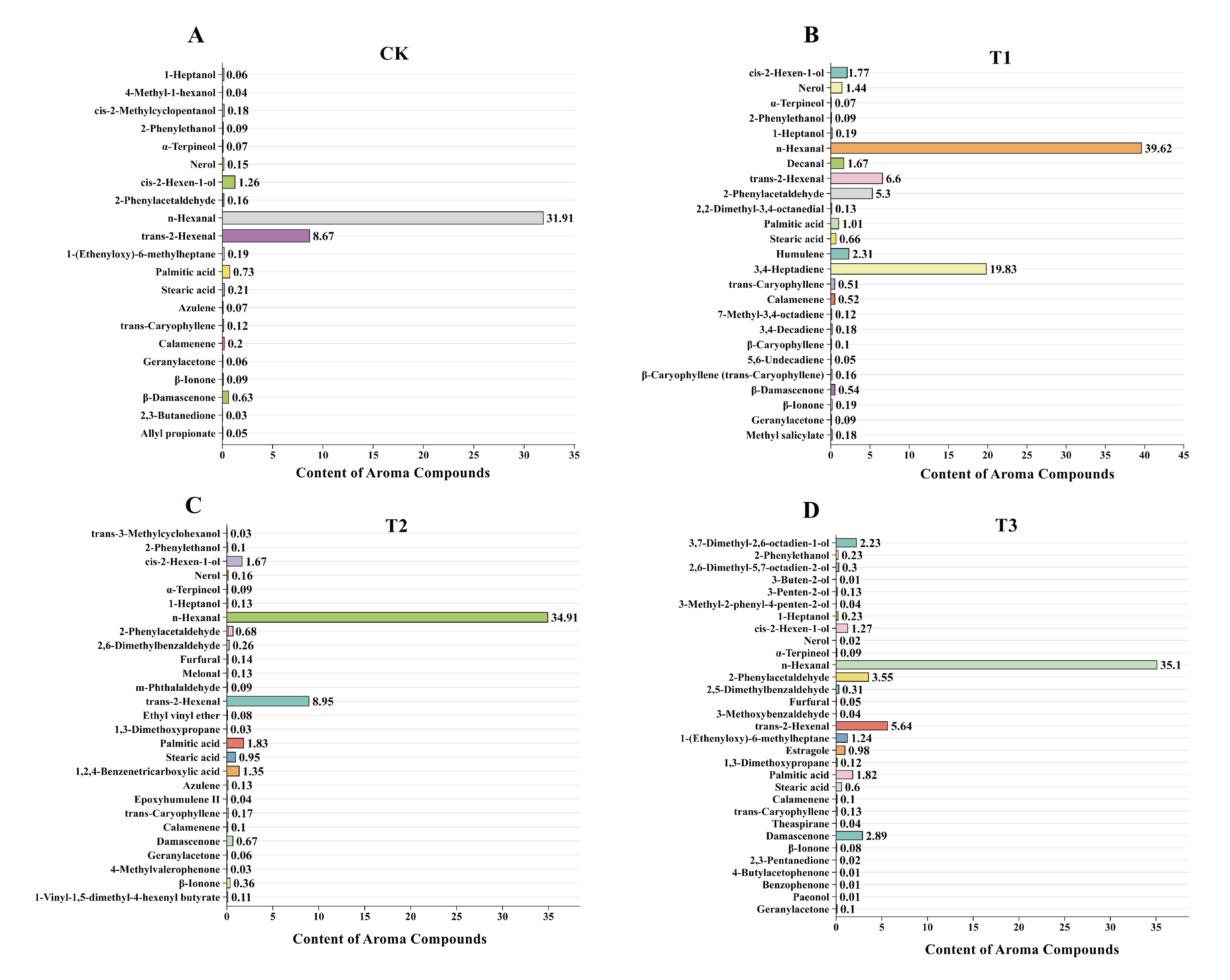

Supplement: Supplementary file 1 [file plants-15-01595-s001.zip › Figure S4.png]

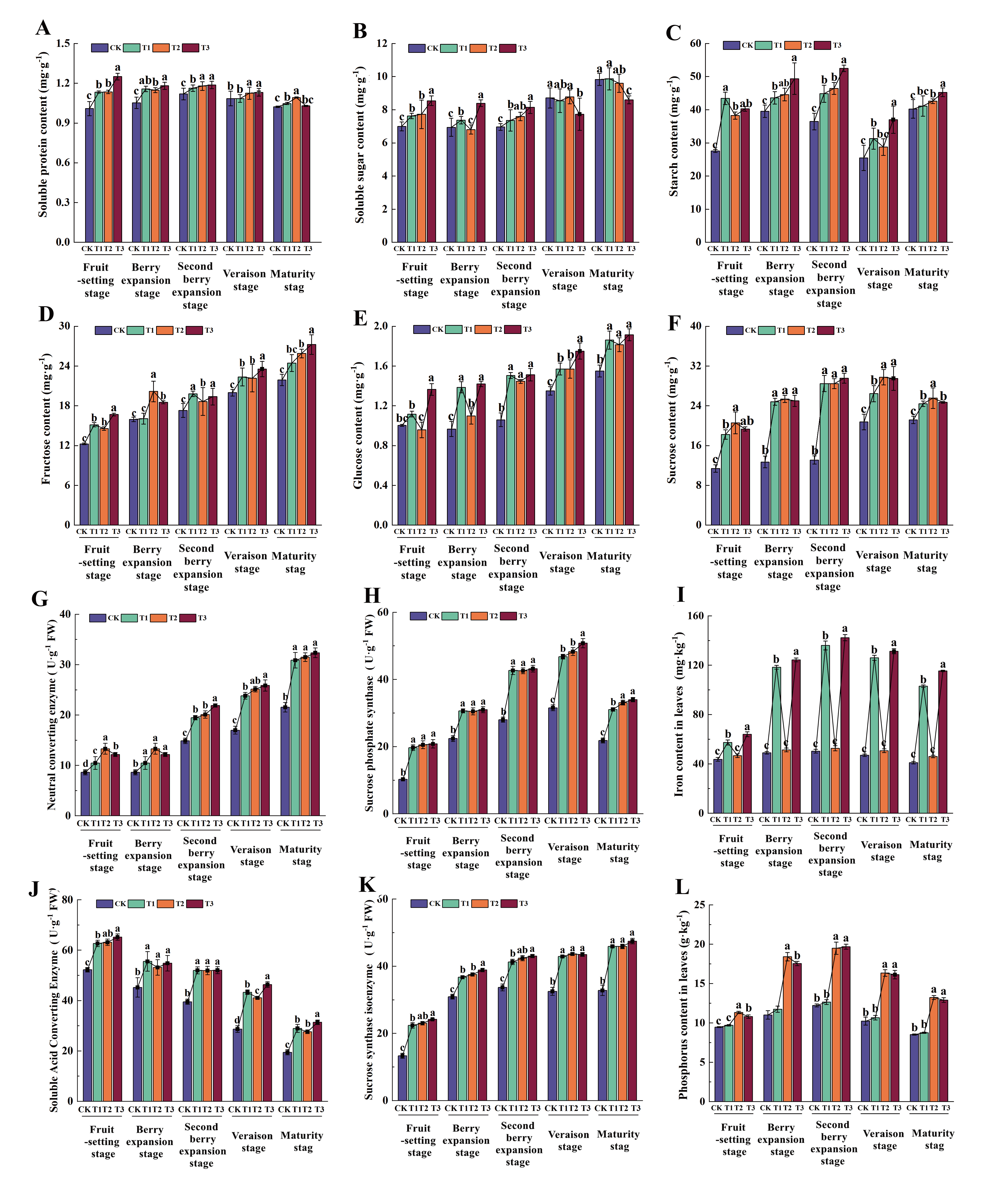

Supplement: Supplementary file 1 [file plants-15-01595-s001.zip › Figure S1.png]

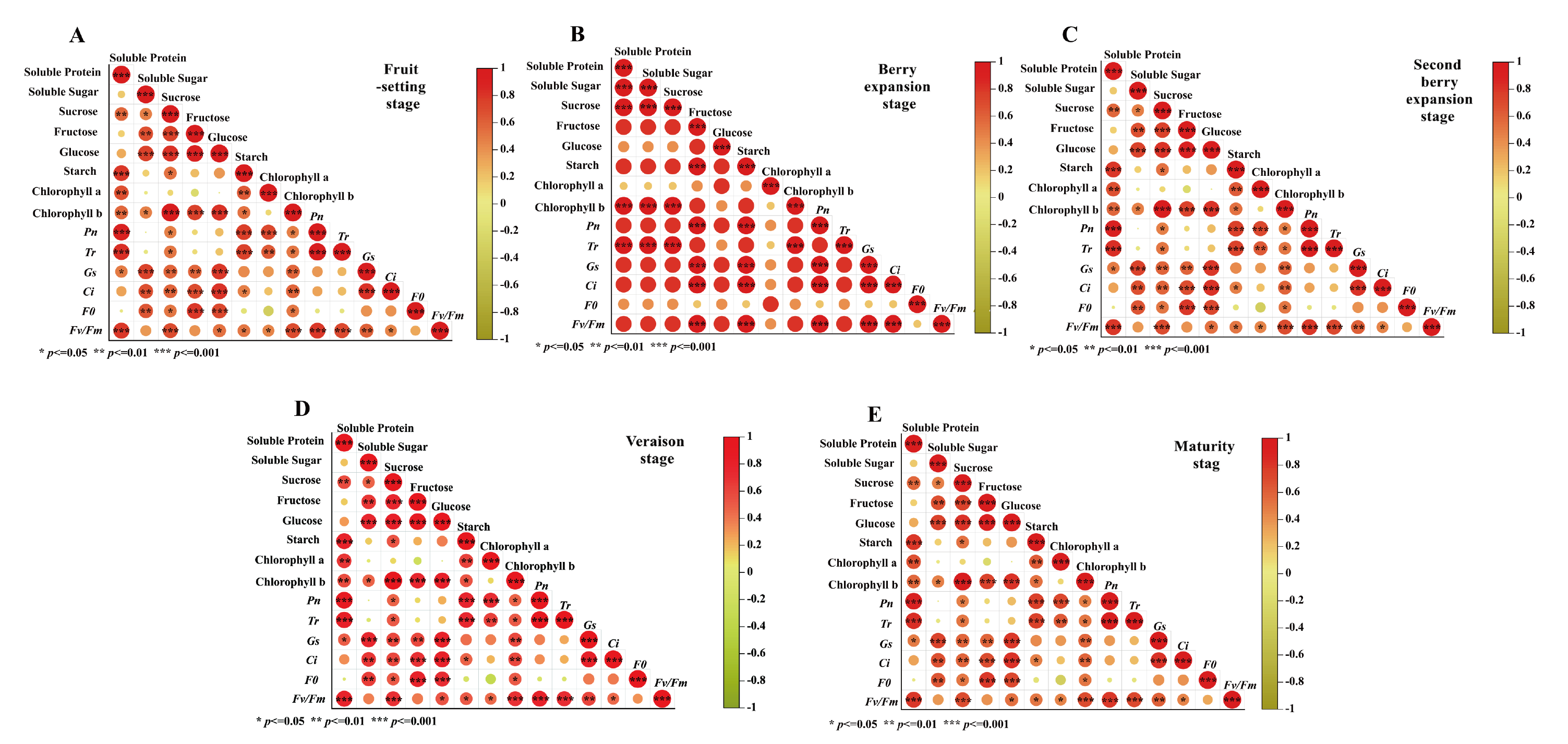

Supplement: Supplementary file 1 [file plants-15-01595-s001.zip › Figure S2.png]

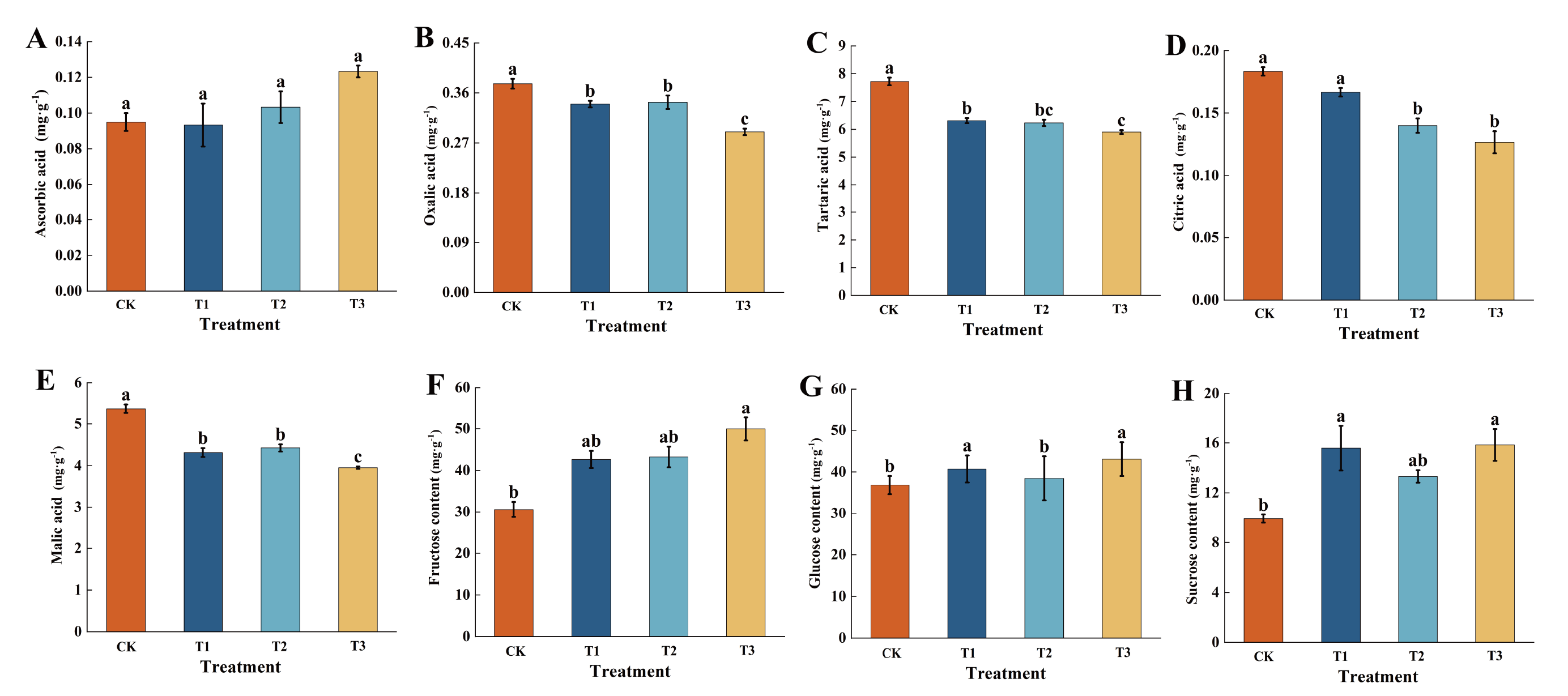

Supplement: Supplementary file 1 [file plants-15-01595-s001.zip › Figure S3.png]
